# Cu(II)@MXene based photothermal hydrogel with antioxidative and antibacterial properties for the infected wounds

**DOI:** 10.3389/fbioe.2023.1308184

**Published:** 2023-11-09

**Authors:** Mingxiang Liu, Lei Zheng, Kangkang Zha, Yayan Yang, Yunping Hu, Kai Chen, Feng Wang, Kunyu Zhang, Wei Liu, Bobin Mi, Xiufeng Xiao, Qian Feng

**Affiliations:** ^1^ Fujian Provincial Key Laboratory of Advanced Materials Oriented Chemical Engineering, College of Chemistry and Materials Science, Fujian Normal University, Fuzhou, China; ^2^ Department of Biochemistry and Molecular Biology, Key Laboratory of Neural and Vascular Biology, Ministry of Education, Hebei Medical University, Shijiazhuang, China; ^3^ Department of Orthopedics, Union Hospital, Tongji Medical College, Huazhong University of Science and Technology, Wuhan, China; ^4^ School of Biomedical Science and Engineering, South China University of Technology, Guangzhou, China; ^5^ Department of Neurosurgery, Renhe Hospital, Huashan North Hospital Baoshan Branch Affiliated to Fudan University, Shanghai, China; ^6^ Key Laboratory of Biorheological Science and Technology, Ministry of Education, College of Bioengineering, Chongqing University, Chongqing, China

**Keywords:** hydrogel, MXene, antibacterial, angiogenesis, wound healing

## Abstract

The regeneration of skin tissue is often impeded by bacterial infection seriously. At the same time, reactive oxygen species (ROS) are often overexpressed in infected skin wounds, causing persistent inflammation that further hinders the skin repair process. All of these make the treatment of infected wounds is still a great challenge in clinic. In this study, we fabricate Cu(II)@MXene photothermal complex based on electrostatic self-assembly between Cu^2+^ and MXene, which are then introduced into a hyaluronic acid (HA) hydrogel to form an antibacterial dressing. The rapid adhesion, self-healing, and injectability of the dressing allows the hydrogel to be easily applied to different wound shapes and to provide long-term wound protection. More importantly, this easily prepared Cu(II)@MXene complex can act as a photothermal antibacterial barrier, ROS scavenger and angiogenesis promoter simultaneously to accelerate the healing rate of infected wounds. Our *in vivo* experiments strongly proved that the inflammatory condition, collagen deposition, vessel formation, and the final wound closure area were all improved by the application of Cu(II)@MXene photothermal hydrogel dressing.

## Introduction

Skin, as the largest organ and the first defense of human body, often suffers from various kinds of injuries (Cui et al., 2022). Fortunately, skin shows a good ability of self-healing if the damages are not serious (Lee et al., 2022). The self-healing of wounds is a complex process, which includes the hemostatic phase, inflammatory phase, proliferative phase, and remodeling phase (Zhao et al., 2019). During the inflammatory phase, ROS secreted by leukocytes, macrophages, and other inflammatory cells prevent bacterial invasion and avoid wound infection, playing an important role in regulating the inflammatory response and the wound healing process (Jin et al., 2020; Sawaya et al., 2020). However, the self-protection of the wounds does not always work to eliminate bacterial infection. Moreover, under long-term bacterial infection, ROS are often overexpressed in the wound to lead the overactivation of proinflammatory cytokines and matrix metalloproteinases (MMPs), thereby disrupting the intracellular homeostasis and prolonging the inflammation phase (Liu and Shi, 2019; Xuan et al., 2021). At the same time, the overexpressed ROS also can damage the endothelial cell and blood vessels (Zhao et al., 2020). All of these create a negative microenvironment to hinder the infected wound healing. Therefore, in order to reverse the negative microenvironment of infected wound, how to remove bacteria and scavenge excessive ROS simultaneously is a research direction which is worth of further pondering.

Until now, for the antibacterial treatment of wounds, antibiotics are still the first choice in clinic (Li et al., 2022a). For example, Suhaeri et al. (2018) loaded ciprofloxacin onto a skin patch for the treatment of infected wounds, and (Fu et al., 2022) introduced gentamicin into a hydrogel network to eliminate bacteria in infected wounds. However, the treatment methods involving loaded antibiotics are often characterized by cumbersome preparation and face challenges such as poor drug permeability, antibiotic resistance risks, and the need for frequent administration (Li et al., 2022b). Therefore, developing new types of antibacterial therapies, such as photothermal therapy (PTT) and photodynamic therapy (PDT), is extremely urgent. Especially, PTT means that the photothermal agent is activated to an excited singlet state by irradiation with near-infrared (NIR) light and subsequently recovered to the ground state by exotherm. The released heat during this process induces the damage to the bacterial membrane, which finally achieve bacterial clearance (Ma et al., 2021). Until now, PTT has received increasing attention because of its broad antibacterial spectrum, non-invasiveness and deep tissue penetration (Zeng et al., 2021). Various two-dimensional (2D) nanomaterials have been reported to be used as photothermal converters for PTT, including graphene or graphene derivatives (Altinbasak et al., 2018), molybdenum disulfide (MoS_2_) (Venkata Subbaiah et al., 2016; Liu et al., 2017), black phosphorus (BP) (Qian et al., 2017; Yang et al., 2018), and transition metal carbides and nitrides (MXenes) (Lin et al., 2017). MXenes is an emerging 2D multifunctional ultra-thin nanomaterial (Li et al., 2017; Murali et al., 2021) with promising applications in biomedical engineering based on its excellent biocompatibility, excellent structural stability, and high photothermal conversion efficiency (Rasool et al., 2016). For example, Wu et al. (2022) developed a MXene photothermal microneedle patch to control the release of the biologic agent IL-17 mAbs through photothermolysis, making it a potential candidate for the treatment of inflamed skin. Li et al. (2018) reported a MXene based composite nanoplatform for efficient synergistic chemotherapy and photothermal therapy for the eradication and prevention of recurrence of hepatocellular carcinoma. More interestingly, MXenes also exhibit satisfactory antioxidative property, so it can scavenge ROS, such as hydrogen peroxide (H_2_O_2_), superoxide radicals (·O_2_^−^), and hydroxyl radicals (·OH) effectively (Feng et al., 2021). For example, Ren et al. (2019) developed MXene protectants that significantly reduced IR-induced ROS production to reverse the damage of the hematopoietic system in irradiated mice. Therefore, MXene-based nanomaterials are actually a promising development direction for the treatment of infected wound. On the other hand, hydrogels, due to the biocompatibility and functionality, are the most rapidly developed wound dressing at present. Firstly, the porous three-dimensional (3D) network structure of hydrogels guarantees their good permeability, allowing the efficient oxygen and nutrient exchange in wound (Qi et al., 2022). At the same time, hydrogels can be easily endowed with special properties, such as injectable and self-healing, and bio-adhesive properties, through the well-design of the hydrogel crosslinking (Liang et al., 2021). Then, these special properties allowed the hydrogels to fill irregularly shaped wound defects easily to provide a lasting physical barrier for wound protection (Li et al., 2022c). Therefore, how to take advantages of 2D nanomaterials and hydrogels to develop the next-generation dressing for infected wound treatment should be an interesting research direction.

**SCHEME 1:**
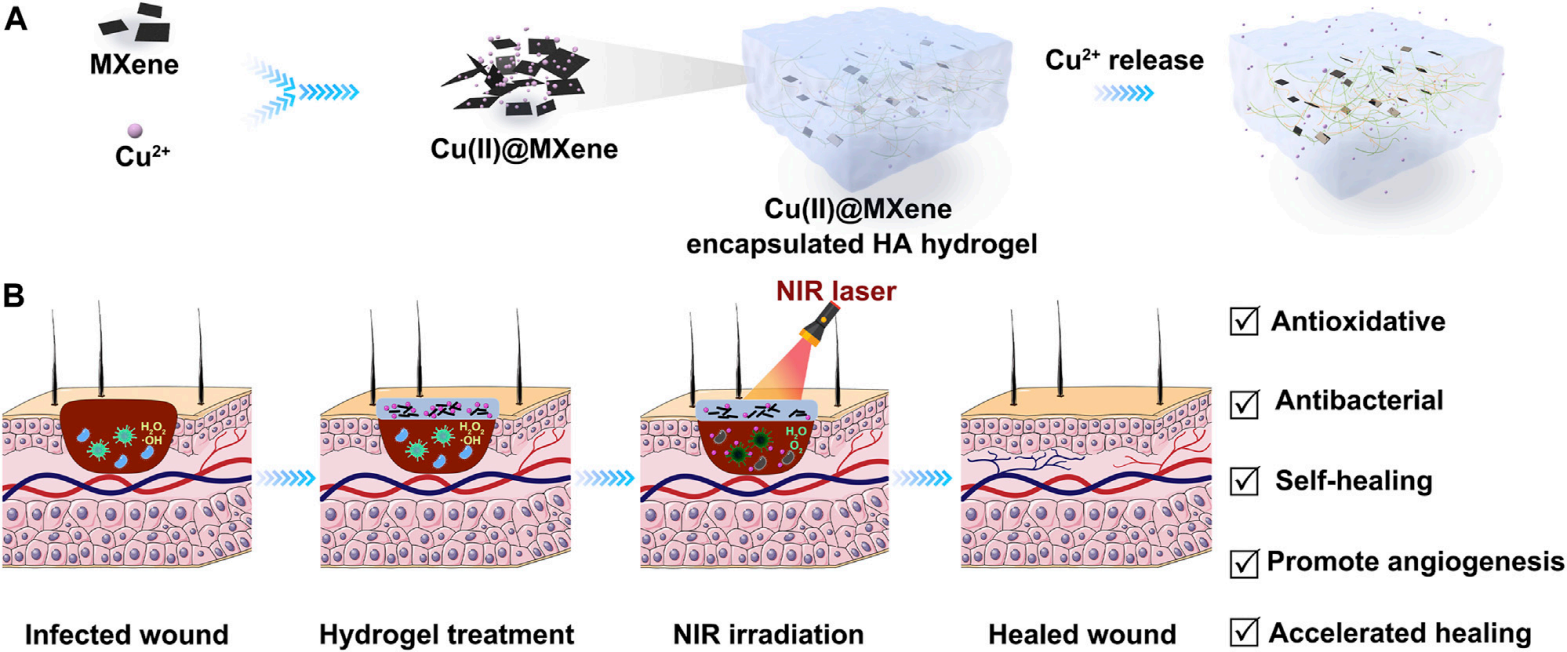
Schematic illustration of the preparation and application of Cu(II)@MXene photothermal hydrogel. **(A)** The Cu(II)@MXene complex were formed through the electrostatic aggregation and the Cu(II)@MXene photothermal hydrogels were prepared by the mixing of Cu(II)@MXene suspension, OHA solution, and HA-ADH solution. **(B)** The Cu(II)@MXene hydrogels could be injected to cover the infected wounds as a protective layer to promote the wound healing.

Herein, we designed a Cu(II)@MXene based photothermal hydrogel system and explored its potential application as a dressing for the infected wound (Scheme 1). The Cu(II)@MXene composite we have designed and prepared can simultaneously act as a photothermal converter and ROS scavenger. Under near-infrared triggering, the composite rapidly releases the loaded Cu^2+^ and exhibits good photothermal synergy with MXene against bacteria. The preparation of Cu(II)@ MXene photothermal hydrogel is simple, without the need for loading antibiotics, reducing the prevalence of drug-resistant pathogens, but its multifunctional characteristics, including injectability, self-healing, antioxidant, antibacterial, and long-term release of Cu^2+^, meet the requirements of infection wound dressings. Our subsequent *in vitro* and *in vivo* studies strongly demonstrated the enormous potential of Cu(II)@ MXene-based photothermal hydrogel in treating infected wounds.

## Materials and methods

### Materials

Sodium hyaluronate (HA, MW: 10 kDa) was purchased from Shandong Focuschem Co., Ltd. (China) Adipic dihydrazide (ADH, 98%), sodium periodate (NaIO_4_, AR), 2-morpholineethanesulfonic acid (MES, 99%), 1-(3-dimethylaminopropyl)-3-ethylcarbodiimide hydrochloride (EDC, 98.5%), copper chloride (CuCl_2_·H_2_O, AR), lithium fluoride (LiF, AR), sodium hydroxide (NaOH, AR), ethylene glycol (EG, AR), hyaluronidase (300 U/mg) were purchased from Shanghai Macklin Biochemical Technology Co. Ltd. (China) Hydrochloric acid (HCl, AR, 37%), hydrogen peroxide (H_2_O_2_, AR, 30%), sulfuric acid (H_2_SO_4_, AR) were bought from Sinopharm Chemical Reagent Co., Ltd. (China) 1-Hydroxybenzotriazole (HOBT) was obtained from J&K Scientific. (China) Titanium aluminum carbide (Ti_3_AlC_2_, 400 mesh) purchased from Adamas Reagent Co. (China) Titanium sulfate (Ti(SO_4_)_2_, AR) was purchased from Shanghai Aladdin Biochemical Technology Co., Ltd. (China).

### Electrostatic aggregation forms Cu(II)@MXene complex

Ti_3_C_2_ MXene was prepared according to previous reported method (Peng et al., 2021). Briefly, 1.6 g of LiF was dissolved in 20 mL of HCl (9 M) to form an etching solution. Then 1 g of Ti_3_AlC_2_ was added to the etching solution and stirring was continued for 24 h. Subsequently, the precipitates were rinsed with deionized water (DI water) and then centrifuged. This process was repeated several times until the pH of the supernatant was >6. Finally, the solid precipitates were continued to be sonicated in DI water for 1 h. The dark green supernatant was collected and stored at 4°C.

MXene suspension (10 mL, 0.5 g/mL) was sonicated and then added to 100 µL of CuCl_2_ solution (5 wt%) and mixed thoroughly with a vortex shaker for 60 s to obtain the suspension of self-assembled Cu(II)@MXene complex (Scheme 1A).

### Synthesis of hexanediyl dihydrazide modified hyaluronic acid (HA-ADH)

Dissolve 1 g of MES in 200 ml of DI water. Adjust the pH of the solution to 6.5 with 1 M NaOH. 2 g of HA was completely dissolved in the MES buffer. Then 2.5 g of EDC and 1.78 g of HOBT were added to the HA solution sequentially. After 1 h of reaction, 9 g of ADH was added into the solution, and then the reaction was continued at room temperature for another 24 h. Finally, the obtained solution was dialyzed against NaCl solution for 3 days and then further dialyzed against DI water for another 3 days (Mw = 14,000). The purified solution was freeze-dried to obtain the final product HA-ADH.

### Synthesis of oxidized hyaluronic acid (OHA)

1 g of HA was completely solved in 100 mL of phosphate buffer solution (PBS) and the solution pH was adjusted to 5.0 with 1 M HCl. 0.5 g of NaIO_4_ was added into the solution and the reaction was continued at room temperature under dark for 5 h. Then 1 mL of EG was added to the solution to terminate the reaction. Finally, the obtained solution was dialyzed against NaCl solution for 3 d, and then further dialyzed against deionized water for 3 days (Mw = 14,000). The purified solution was freeze-dried to obtain OHA.

### Preparation of hydrogels

HA-ADH and OHA were dissolved in PBS (pH = 7.4), respectively. The HA hydrogel was prepared by the mixing of HA-ADH and OHA solution with specific concentration. The concentration of HA-ADH solution was fixed at 4 (w/v)% and the concentration of OHA solution was ranged from 0.6, 0.8, 1.0 to 1.2 (w/v)%. For the preparation of the Cu(II)@MXene hydrogel, the Cu(II)@MXene suspension was added directly to the aforementioned mixing process.at concentrations of 30, 90, and 150 μg/mL.

### Chemical structure characterization

The microstructure of MXene was characterized by transmission electron microscopy (TEM, Tecnai F20, FEI, United States), atomic force microscopy (AFM, Dimension ICON, Bruker, Germany). The particle size distribution and zeta potential of MXene were characterized by laser particle sizer (Nano ZSE, Malvern, United Kingdom) at 25°C. Each test was repeated three times. The crystal structure of MXene was characterized by X-ray diffractometry (XRD, X'Pert PXRD, PANalytical B.V., Netherlands). The molecular structure of MXene was characterized by X-ray photoelectron spectroscopy (XPS, ESCALAB Xi+, Thermo Fisher Scientific, United States) and Raman spectroscopy (Raman, LabRAM HR Evolution, HORIBA, Japan). The Cu^2+^ loading and releasing ability of Cu(II)@MXene complex were analyzed with an inductively coupled plasma spetrometer (ICP, Optima8000, Perkin Elmer, Singapore). The ability of MXene to scavenge ROS was evaluated by enzymatic calibrator (Multiskan Sky, Thermo Fisher Scientific, United States). Structural analysis of HA-ADH, OHA was performed by nuclear magnetic resonance spectrometry (NMR, AVANCE 400, Bruker, Germany). The coating formation and microstructure of the hydrogels were characterized by Fourier transform infrared spectroscopy (FT-IR, Nicolet IS50, Thermo Fisher Scientific, United States), scanning electron microscopy (SEM, Regulus 8100, HITACHI, Japan). The hydrogel samples were rapidly frozen in liquid nitrogen for 5 min and immediately freeze-dried for 48 h to remove moisture. The samples were plated by sputtering for 30 s.

### *In vitro* photothermal effect of Cu(II)@MXene complex

The photothermal properties of Cu(II)@MXene complex were investigated by exposing NIR with a wavelength of 808 nm. Briefly, Cu(II)@MXene suspension with different concentration (30, 90, 150 μg/mL) were loaded in 1.5 mL centrifuge tubes and the solutions were irradiated with NIR with different power (0.5, 1.0, 1.3, 1.8 W/cm^2^). The temperature change of the solution was then recorded with a temperature sensing recorder until the temperature stopped rising. The photothermal properties of the materials were evaluated by using temperature-time curves.

### Swelling properties

Firstly, hydrogel samples were placed in centrifuge tubes containing 500 µL of PBS buffer (pH = 7.4, 37°C) to simulate physiological conditions. After that, the hydrogels were periodically removed, and after wiping the surface water with filter paper, the mass of the hydrogels was weighed, and the above process was repeated until the mass of the hydrogels was stable. Finally, the swelling rate of the hydrogel was calculated based on the swelling mass. The experiment was repeated three times for each sample and the average value was calculated. The swelling rate was calculated by the following equation: SR=(W_t_-W_0_)/W_0_×100%, where SR-swelling rate; W_t_-weight of hydrogel after specific time swelling; W_0_-weight of initial hydrogel.

### Biodegradation behavior

The biodegradation behavior of hydrogels in the wet state after reaching solubilization equilibrium was determined experimentally. Briefly, the hydrogels were placed in a PBS buffer containing hyaluronidase (enzyme concentration of 100 U/mL, pH = 7.4, 37°C) and replaced with fresh PBS buffer of hyaluronidase every 2 days to simulate physiological conditions. After that, the hydrogels were periodically removed and the mass of the hydrogels was weighed after wiping the surface water with filter paper, and the process was repeated until the hydrogels were completely biodegraded. Finally, the biodegradation rate of the hydrogel was calculated based on the residual amount of the hydrogel. The experiment was repeated three times for each sample and the average value was calculated. The biodegradation rate was calculated by the following equation: DR=(W_s_-W_d_)/W_s_×100%, where, DR-biodegradation rate; W_d_-weight of hydrogel after a specific time of biodegradation; W_s_-weight of hydrogel with dissolution equilibrium.

### Rheological performance characterization

The rheological properties of the hydrogels were evaluated using a TA rheometer (DHR 2, Waters, United States). Parallel plates of 8 mm diameter with a fixed gap size of 1 mm were used at 25°C. The time sweep at strain of 1% and a frequency of 0.1 Hz. Then strain sweep was performed at a frequency of 1 Hz and a strain range of 1%–600%. As for the shear thinning experiments, the hydrogels were subjected to alternating high strain (500%) and low strain (1%) with a time interval of 60 s.

### Ion loading and release capacity testing

The Cu^2+^ loading efficiency was determined by measuring the Cu^2+^ content in the supernatant after Cu(II)@MXene self-assembly. The experiment was repeated three times for each sample and the average value was calculated. The loading rate was calculated as follows: LR=(C_b_-C_a_)/C_b_×100%, where LR-loading rate of Cu^2+^; C_b_-the beginning concentration of Cu^2+^; C_a_-the final concentration of Cu^2+^ in the supernatant.

The ion release property of Cu(II)@MXene hydrogel was similarly investigated. Briefly, 80 μL of Cu(II)@MXene hydrogel was incubated in 1 mL of DI water at 37°C. The concentration of Cu^2+^ in the supernatant was measured at different time points. In addition, to investigate the effect of laser irradiation on the ion release properties of the hydrogels, NIR laser irradiation using a laser power density of 1.5 W/cm^2^ was added for 10 min at 16 h. The experiment was repeated three times for each sample and the mean value was calculated.

### Evaluation of reactive oxygen scavenging capacity

The ROS scavenging ability of the hydrogels was evaluated by the reaction of Ti(SO_4_)_2_ with hydrogen peroxide to form a complex. Briefly, and 80 μL of Cu(II)@Mxene hydrogel was immersed into 0.5 mL of the assay solution (2 M H_2_SO_4_ and 5 wt% Ti(SO_4_)_2_ and 1 mM H_2_O_2_). At different time points, the supernatant (180 μL) was collected and the absorbance at 408 nm was measured by an enzyme marker, and the ability of the hydrogel to scavenge reactive oxygen species was evaluated by calculating the concentration of H_2_O_2_ in the solution.

### *In vitro* cytocompatibility

L929 cells were seeded onto 24-well plates at 1 × 10^4^ cells/well and cultured in DMEM/F12 supplemented with 10% FBS in 5% CO_2_ at 37°C. Then, 100 μL PBS or sterile hydrogel was added into 24-well plates for another culture of 24 h. As for Cu(II)@MXene hydrogel-150+NIR group, the NIR was set as 1.5 W/cm^2^ 808 nm laser for 5 min. A CCK-8 kit (Dojindo, Kumamoto, Japan) was used to evaluate the proliferation rates of L929 cells treated by co-culturing with PBS or hydrogel at 24 h. The optical density (OD) value (absorbance at 450 nm) was measured with a plate reader. A Calcein-FITC/PI live-dead staining kit (Solarbio, Beijing, China) was used to evaluate the viability of L929 cells after treatment with PBS or hydrogels for 24 h. Images were obtained using a fluorescence microscope (Olympus).

### Tube formation assay

HUVECs were treated with the extracts of PBS, HA hydrogel, Cu(II)@MXene for 24 h. Then, the cells (2 × 10^4^/well) were cultured in 96-well plates pre-coated with Matrigel for 6 h. Three randomly-chosen fields were photographed using an inverted microscope (Olympus, Tokyo, Japan) and the branch points and tube length were determined by ImageJ software.

### *In vitro* antibacterial properties

100 μL of hydrogel was co-cultured with 50 μL of bacterial solution with a concentration of 105 CFU/mL to the 96-well plate (the group without hydrogel is the positive control group). No NIR treatment for 5 min and NIR treatment (1.5 W/cm^2^, 5 min) were given, respectively. Subsequently, 10 μL of treated bacterial solution was mixed with another 90 uL of Mueller-Hinton Broth (MHB) bacterial solution was added and incubated in 96-well plates for 12 h. The absorbance at 600 nm was then read using an enzyme marker. At the same time, another 10 μL of treated bacterial solution was resuspended in 1 mL of PBS and plated on LB agar for another 12 h of culture before taking pictures.

### *In vivo* wound healing

The C57BL/6 male mice were anesthetized via intraperitoneal injection of 2% pentobarbital sodium (50 mg/kg; Sigma Aldrich) and full-thickness round skin wounds (d = 1 cm) were made by a sharp round pouch. Each wound was infected with *S. aureus* suspension (30 μL) with 3 × 10^8^ CFU/mL for 10 min. The C57BL/6 mice were randomly divided into 5 groups and treated with 200 μL of PBS, HA hydrogel, and Cu(II)@MXene hydrogel, respectively (*n* = 5). Among them, a group of Cu(II)@MXene hydrogel were irradiated with 1.5 W/cm^2^ 808 nm laser for 5 min. The wounds were bandaged with gauze and were photographed on days 0, 3, 7, and 14 d.

### Histological analysis

Wound sections from rats were stained with hematoxylin and eosin (H&E), Masson and CD31, and wound healing mechanisms were analyzed under standard procedures.

### Statistical analysis

Pair groups were assessed with Student’s t-test, while multiple group comparisons were performed with a one-way analysis of variance (ANOVA) with Tukey’s *post hoc* test.

## Results and discussion

### Characterization of MXene nanosheets

**FIGURE 1:**
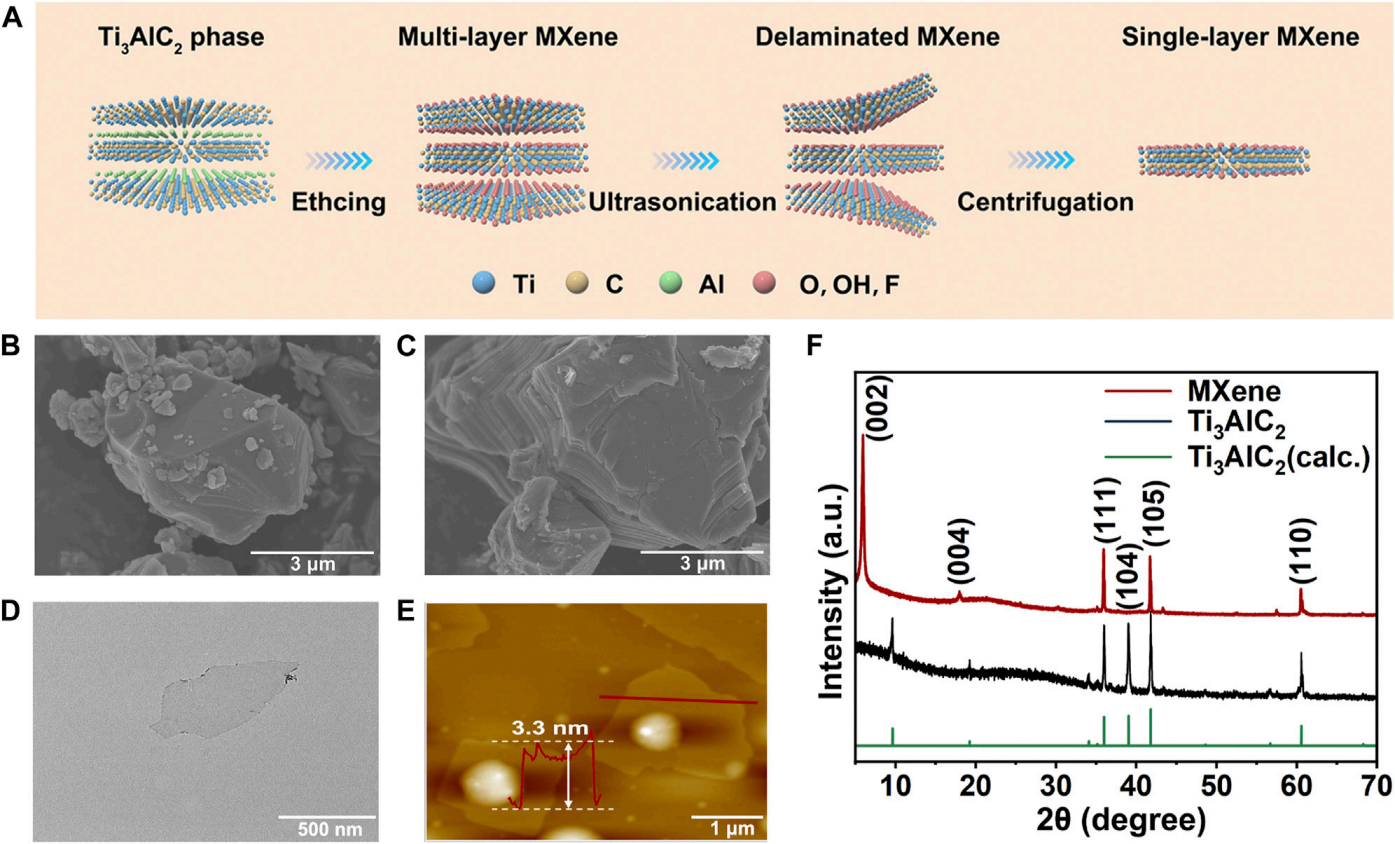
Synthesis and characterization of MXene nanosheets. **(A)** Schematic diagram of the synthesis process of 2D MXene nanosheets. SEM images of **(B)** Ti_3_AlC_2_ (MAX) phase and **(C)** multi-layer MXene. **(D)** TEM image of single-layer MXene. **(E)** AFM image of single-layer MXene. **(F)** XRD spectra of single-layer MXene and Ti_3_AlC_2_ powder.

The single-layer MXene was prepared by a classical liquid phase exfoliation and ultrasonic layering strategy for the MAX phase (Ti_3_AlC_2_), as shown in Figure 1A. The SEM images clearly demonstrated the etching progress from bulk MAX phase (Figure 1B) to multi-layer MXene (Figure 1C). Then, single-layer MXene was successful obatain via ultrasound exfoliation of multi-layer MXene as shown in TEM (Figure 1D) and AFM (Figure 1E) images. Both of these results show that the final exfoliated MXene nanosheets had an ultrathin and transparent structure with an average thickness of about 4 nm and lateral dimensions of about 150–300 nm (Supplementary Figure S1A). The phase composition of single-layer MXene was further analyzed by XRD (Figure 1F). Compared to the raw material MAX, the single-layered MXene showed a disappearance of the (104) characteristic peak, a left-shift of the (002) peak, and a widening of the (002) peak (Feng et al., 2017). All of these indicated that the Al phase was stripped, confirming the successful etching of MAX to produce MXene. Raman spectroscopy measurements (Supplementary Figure S2) were used to investigate structural defects and vibrational modes in the prepared samples. In the case of Ti_3_AlC_2_, a characteristic peak of out-of-plane stretching vibrations of Ti and C atoms was clearly observed at 271 cm^−1^. After etching, this characteristic peak shifted to 202 cm^−1^. The overall vibrational mode was significantly broadened due to the structural changes induced by the etching progress (Sarycheva and Gogotsi, 2020), confirming the results in agreement with the X-ray diffraction analysis. For the following experiments, we used MXene to represent single-layered MXene.

### Characterization of Cu(II)@MXene complex

**FIGURE 2:**
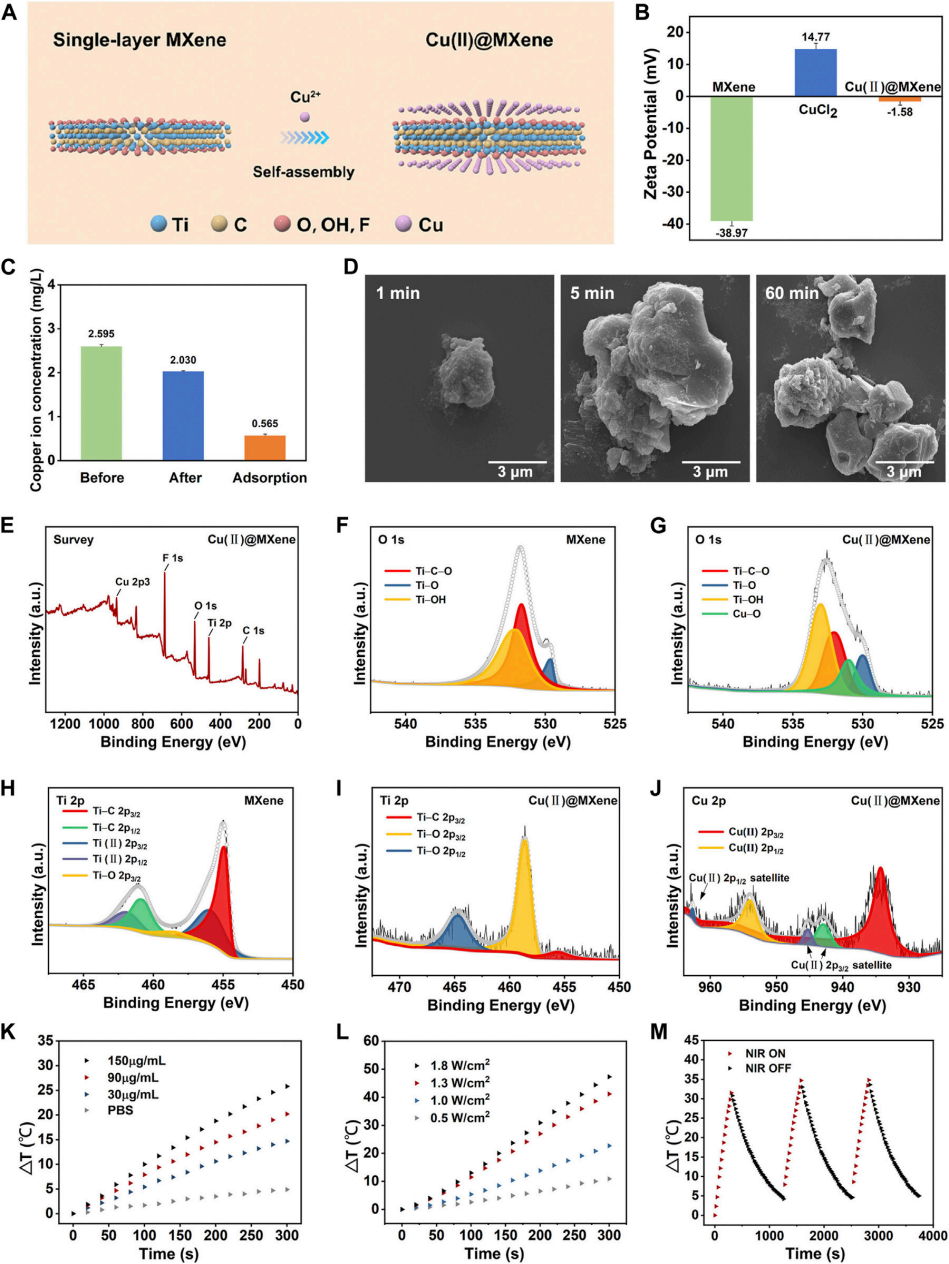
Synthesis and characterization of Cu(II)@MXene complex. **(A)** Schematic diagram of the synthesis process of Cu(II)@MXene complex. **(B)** Zeta potential of MXene, CuCl_2_, and Cu(II)@MXene. **(C)** Concentration of Cu^2+^ in the supernatant before and after electrostatic self-assemble between Cu^2+^ and MXene. **(D)** The SEM images of Cu(II)@MXene at different time points. XPS spectral analysis, measured spectra of **(E)** Cu(II)@MXene, **(F)** O 1s of MXene, **(G)** Cu(II)@MXene, **(H)** Ti 2p of MXene **(I)** Cu(II)@MXene, and **(J)** Cu 2p of Cu(II)@MXene. **(K)** Photothermal conversion efficiency of the Cu(II)@MXene suspension with different concentrations under NIR irradiation with 1.0 W/cm^2^. **(L)** The thermal increase curves of the Cu(II)@MXene suspension (150 μg/mL) under NIR irradiation with different power densities. **(M)** The recycling heating profile of Cu(II)@MXene suspension (150 μg/mL) under NIR light irradiation (1.0 W/cm^2^) for three on/off cycles.

The 2D layered planar structure provides MXenes with abundant anchor points and an extremely high specific surface area. After etching by LiF-HCl solution, the MXene surface is distributed with a large number of negatively charged groups (-OH, -O and -F), which can serve as available sites for trapping cations. Due to the strong adsorption affinity of Ti-O and Ti-OH for metal ions, the positively charged Cu^2+^ could undergo the ion-exchange reactions with the negatively charged groups (-OH, -O) on the MXene surface, forming Cu(II)@MXene complex (Figure 2A). This self-assembly progress between Cu^2+^ and MXene could be observed by TEM (Supplementary Figure S3) and also proved by the Zeta potential change in Figure 2B. The pure MXene suspension exhibited a negative zeta potential (−38.9 mV) due to the strongly negatively charged groups in the surface, while the zeta potential of Cu(II)@MXene dramatically increased to a value (−1.5 mV) which was close to neutral because of the absorption of Cu^2+^ in the surface of MXene (Yin et al., 2020). In order to test the Cu^2+^ absorption efficiency, we tested the Cu^2+^ concentration in the supernatant before and after the Cu(II)@MXene formation as shown in Figure 2C. The loading efficiency of Cu^2+^ in the surface of MXene was about 22%. The SEM images (Figure 2D) showed that the self-assembly among Cu^2+^ and single-layer MXene rapidly occurred within the first 1 min. The EDS image (Supplementary Figure S4) indicates an effective binding between Cu^2+^ and MXene. The morphology of Cu(II)@MXene maintained stable at micron level, which was consistent with the dynamic light scattering (DLS) result (Supplementary Figure S1B). Although Cu(II)@MXene exhibited a certain degree of aggregation over time, simple shaking could relieve the aggregation effectively (Supplementary Figure S5). Therefore, we believed that the dispersibility and stability of the Cu(II)@MXene could meet our requirements for the wound dressing preparation. To understand the elemental composition and molecular structure of the complex, we used X-ray photoelectron spectroscopy (XPS) to further analyze MXene and Cu(II)@MXene. As for MXene, there was no obvious peak of Al, further indicating that the aluminum layer was effectively stripped (Supplementary Figure S6A), while the C 1s peak indicated that the primary structure of MXene was not damaged during the etching process (Supplementary Figure S6C). The characteristic Cu 2p3 peak can be easily observed for Cu(II)@MXene, strongly proving the successful Cu^2+^ doping in the surface of MXene (Figure 2E). Compared to the O 1s spectrum of Mxene (Figure 2F), except the similar 529.6, 531.7, and 532.1 eV peaks which were assigned to Ti-O, Ti-C-O and Ti-OH (Song et al., 2021), respectively, there was another newly appeared characteristic peak on 530.9 eV in the O 1s spectrum of Cu(II)@MXene associated with Cu-O (Figure 2G), confirming the adsorption of Cu^2+^ in the MXene surface. As shown in the Ti 2p spectrum of MXene (Figure 2H), five peaks located at 454.9, 460.8, 455.9, 461.9, and 458.5 eV correspond to Ti-C 2p_3/2_, Ti-C 2p_1/2_, Ti(II) 2p_3/2_, Ti(II) 2p_1/2_ and Ti-O 2p_3/2_, respectively (Zhang et al., 2020a). After introduction of Cu^2+^, the peak of Ti-O 2p_3/2_ shifted to higher energy values and were significantly enhanced (Figure 2I), which indicated the interaction between Cu^2+^ and the oxygen-containing groups of MXene to form Cu-O bonds. The presence of Cu^2+^ was also further verified by the strong satellite lines located at 962.6 eV and between 940 eV and 946 eV besides the two main peaks at 934.5 and 954.4 eV in Figure 2J (Xu et al., 2022). All of the above-mentioned data strongly evidenced the successful fabrication of Cu(II)@MXene.

To investigate the photothermal conversion efficiency of the Cu(II)@MXene complex, the Cu(II)@MXene suspension with different concentration was exposed to 808 nm NIR irradiation with different power for 5 min. The results proved that the heating behavior of Cu(II)@MXene complex could be accelerated by increasing the suspension concentration (Figure 2K) and NIR power (Figure 2L). The real-time heating of Cu(II)@MXene under NIR was also visually reported by the thermal images in Supplementary Figure S7. All of these results demonstrated that the Cu(II)@MXene had an outstanding photothermal conversion efficiency. Compared to some previous studies about PTT nanomaterials for biotherapeutic use (Jiang et al., 2020; Ni et al., 2020), our Cu(II)@MXene complex could reach to a rapid heating behavior to 70°C under a such low concentration of 150 μg/mL and a mild NIR power of 1.8 W/cm^2^ within 5 min. Based on the all above results, we chose Cu(II)@MXene concentration of 150 μg/mL and NIR power of 1.8 W/cm^2^ for the following experiments. The heating cycle test (Figure 2M) showed the photothermal stability of the Cu(II)@MXene complex, indicating the cyclic PTT potential for the infected wounds of our Cu(II)@MXene based composite hydrogel wound dressing.

### Synthesis and characterization of Cu(II)@MXene photothermal hydrogels

**FIGURE 3:**
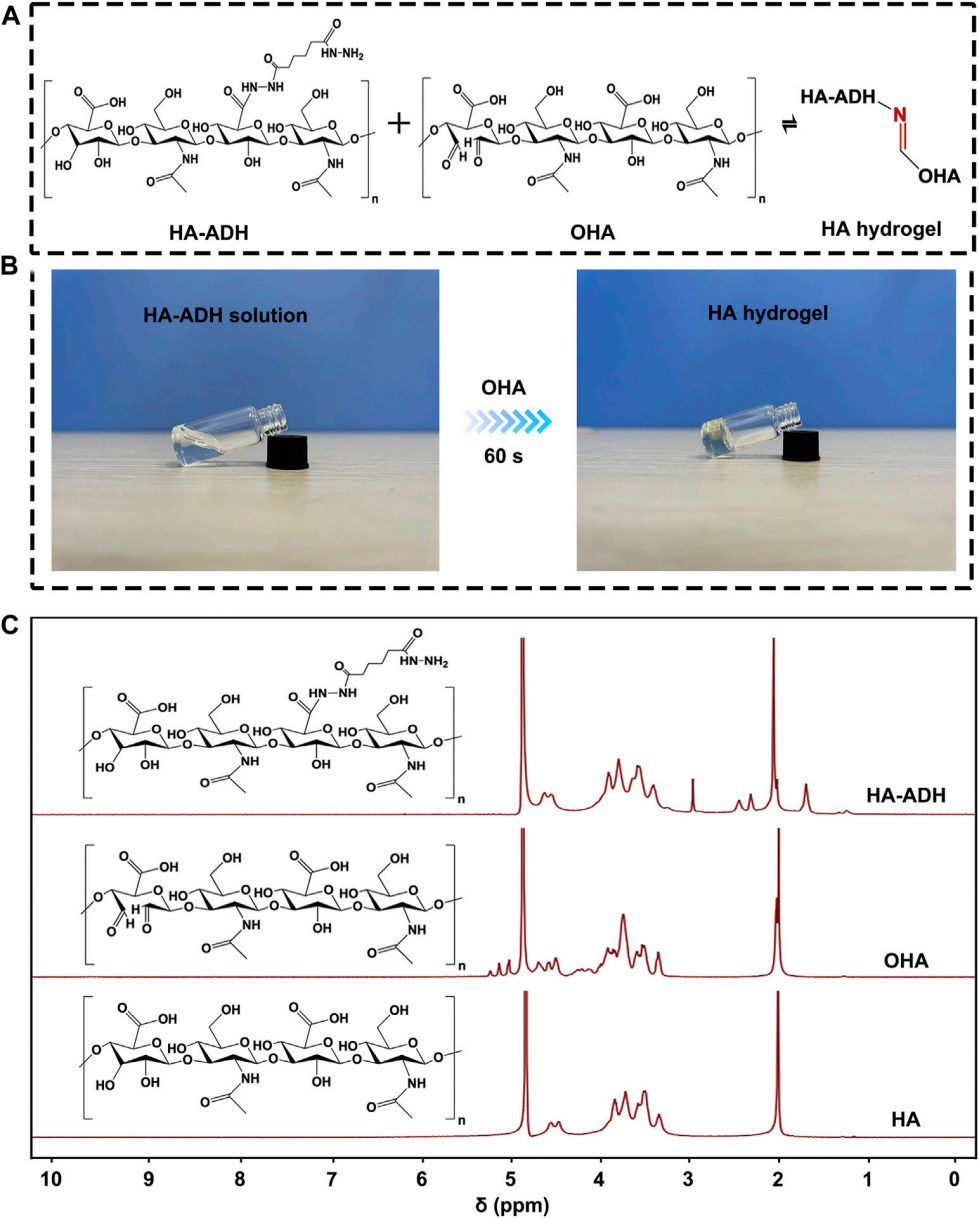
The preparation of HA hydrogel. **(A)** The crosslinking formation between HA-ADH and OHA inside the HA hydrogel. **(B)** Photograph of HA hydrogel formation by an equal volume mixing of OHA and HA-ADH solution at room temperature. **(C)** ^1^H NMR of HA, OHA, and HA-ADH.

We chose HA hydrogel formed with Schiff base reaction as the main scaffold for our Cu(II)@MXene photothermal system (Figure 3A). Therefore, the formed hydrazone crosslinking could meet the injectable, self-healing, and bio-adhesive requirements of wound dressing. As a natural polysaccharide inside the human body, HA possesses excellent biocompatibility and moisture absorption. Moreover, hyaluronic acid is capable of promoting wound healing through facilitating cell migration and mediating cell signaling. Before the HA hydrogel formation, HA should be modified to OHA and HA-ADH, respectively (Supplementary Figure S8). The successful synthesis of OHA and HA-ADH were proved by ^1^H NMR (Figure 3B) and FT-IR (Supplementary Figure S9A). Compared to pure HA, we could observe the methylene proton peaks in HA-ADH (1.66 ppm, 2.27–2.40 ppm) and the hemiacetal peaks (4.94–5.15 ppm) in OHA (Xu et al., 2017). These results were consistent with the FT-IR results (Supplementary Figure S9A) (Liu et al., 2022). Especially, the disappearance of the carbonyl peak at 1,735 cm^−1^ in the FT-IR spectrum of HA hydrogel indicated the Schiff base reaction between the aldehyde groups of OHA and the hydrazide groups of HA-ADH (Supplementary Figure S9B). Of course, the photographs in Figure 3C further demonstrated that the Schiff base reaction between OHA and HA-ADH could guarantee the HA hydrogel fabrication by one-step equal volume mixing of OHA and HA-ADH solution.

**FIGURE 4:**
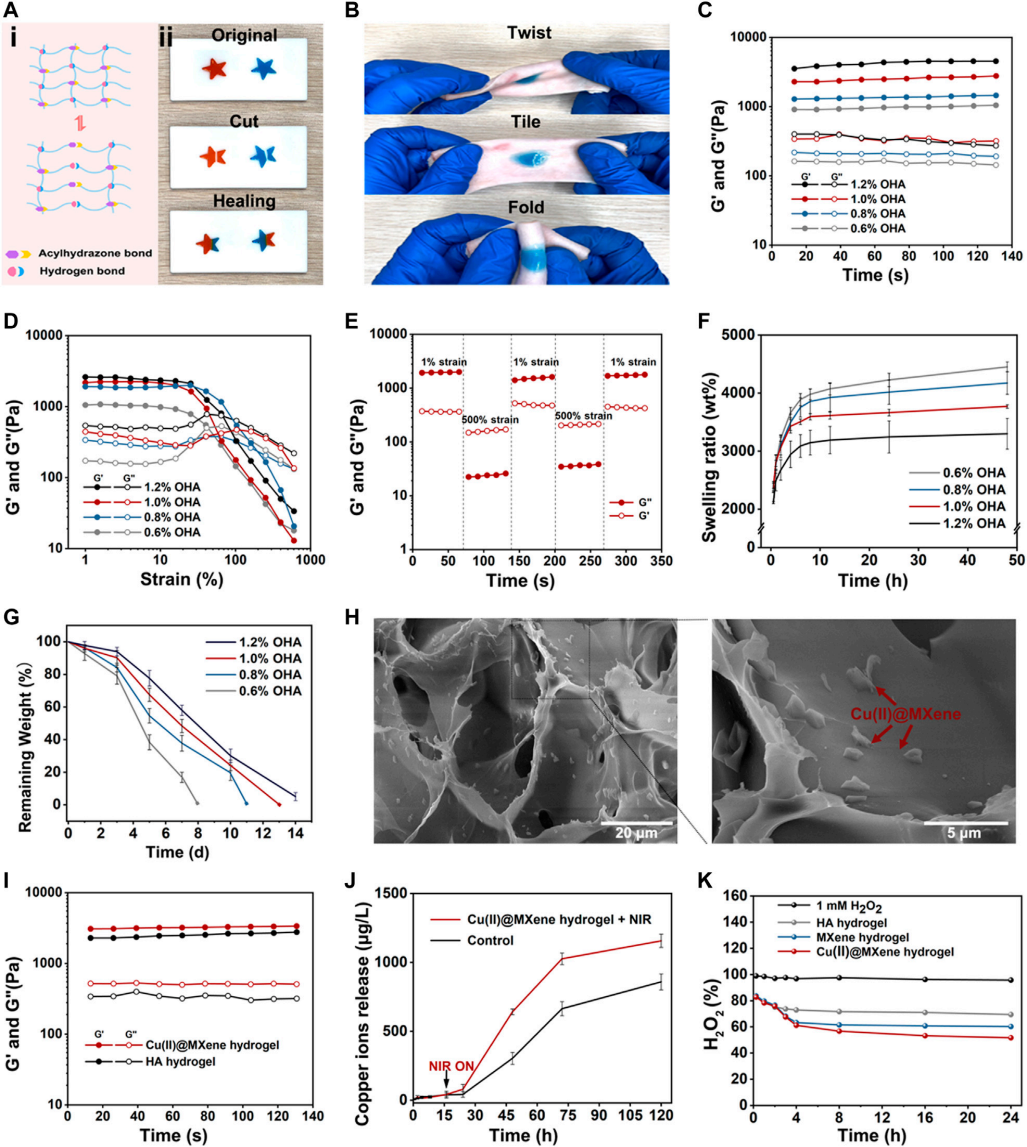
The characterizations of HA hydrogel and Cu(II)@MXene hydrogel. The **(A)** self-healing and **(B)** bio-adhesive properties of HA hydrogel. The rheological **(C)** time sweep and **(D)** strain sweep of HA hydrogel. **(E)** The time sweep of HA hydrogel under cyclic low-high strain. **(F)** Swelling properties of HA hydrogels in PBS. **(G)** *In vitro* degradation property of HA hydrogels in PBS under 37°C. **(H)** Cu(II)@MXene hydrogel and local magnified SEM images. **(I)** The rheological time sweep test comparison between HA hydrogel and Cu(II)@MXene hydrogel. The HA hydrogel groups was differentiated by the concentration of OHA. **(J)** Cu^2+^ release curves of Cu(II)@MXene hydrogels with and without NIR irradiation. **(K)** The ROS scavenging efficiency curve of Cu(II)@MXene hydrogel.

Thanks to the rapid Schiff base reaction between of OHA and HA-ADH, this HA hydrogel could be formed by a dual-syringe model injection as shown in Supplementary Figure S10 and Supplementary Video S1. In this way, this HA hydrogel could realize satisfactory covering for wounds of different shapes. Furthermore, as the hydrazone crosslinking of HA hydrogel was a kind of dynamic covalent bond (Yang et al., 2020), this HA hydrogel also had a good self-healing property (Figure 4A), which was conducive to keeping the integrity of the HA hydrogel to prolong the serves time of our Cu(II)@MXene wound dressing. Moreover, the aldehyde groups of OHA could react with the amino groups of the host tissue to reach the tight adhesion of the HA hydrogel to tissue (Figure 4B). This bio-adhesive property could further guarantee the effective protection of our designed Cu(II)@MXene based dressing for the infected wounds.

We further evaluated the viscoelastic properties of the HA hydrogels by rheological tests. In Figure 4C, the time sweep results of HA hydrogels showed that for all groups the storage modulus (G′) was higher than the loss modulus (G″), indicating the stable state of HA hydrogels. With the OHA/HA-ADH ratio increased, the G′ of the hydrogels subsequently increased from 900 to 3,500 Pa. This phenomenon indicated that the crosslinking density of HA hydrogel increased with the increase of the OHA content. The strain sweep results (Figure 4D) showed that the intersection of G′ and G″ which indicated the hydrogel fracture, was at about 100% strain for all HA hydrogel groups. Based on this data, we chose a high strain at 500% and a low strain at 1% to test the shear-thinning property of our HA hydrogel. Figure 4E demonstrated that the HA hydrogel had a rapid conversion between the “sol” (high strain) and “gel” (low strain) state, which was convincing evidence for the reversibility of the hydrazone crosslinking of the HA hydrogel. This was also the fundamental for the self-healing property of the HA hydrogel. We also observed the *in vitro* swelling (Figure 4F) and degradation (Figure 4G) behavior of the HA hydrogel. After 8 h, all the HA hydrogel groups could reach to the equilibrium swelling (Supplementary Figure S11) and the equilibrium swelling ratio presented a downward trend with the increase of the OHA content. Meanwhile, the changing of the degradation speed of HA hydrogel also presented a similar trend as that of the swelling ratio. These phenomena were due to the increase of crosslinking density with the increase of OHA content. Considering that the difference between 1% and 1.2% group was not significant, we chose 1% group as the substrate materials to be assembled with the prepared Cu(II)@MXene complex to form the final photothermal hydrogel system. In the SEM images of the Cu(II)@MXene hydrogel (Figure 4H), we could easily observe the aggregation of the doped Cu(II)@MXene nanosheets inside the network of the HA hydrogel. The EDS elemental mapping of Cu^2+^ further illustrated the uniform distribution of the Cu(II)@MXene nanosheets inside hydrogel. The further time sweep comparison between the HA hydrogel and the Cu(II)@MXene hydrogel demonstrated that the addition of Cu(II)@MXene had a positive effect on the modulus of hydrogel (Figure 4I). We speculated that it was related to the formation of hydrogen bonds inside the Cu(II)@MXene hydrogel (Zhang et al., 2020b). FT-IR spectra showed that a newly appeared absorption peak at 1,703 cm^−1^ in Cu(II)@MXene hydrogel which was related to the ν_C=O_ stretching vibration of the carboxyl group (Supplementary Figure S9B). This might represent the hydrogen bonds formed between the carboxyl group of HA backbones and the surface of Cu(II)@MXene complex.

The Cu^2+^ released from the Cu(II)@ MXene photothermal hydrogel system has a good synergistic antibacterial effect (Mitra et al., 2020). In addition, the long-term release of Cu^2+^ promotes blood vessel regeneration and collagen deposition (Xiao et al., 2017; Xiao et al., 2018). The results demonstrated that NIR irradiation could speed up the Cu^2+^ release (Figure 4J). This should be due to the accelerated Cu^2+^ motion induced by the increase temperature under NIR irradiation. Under the NIR condition, the sustained Cu^2+^ release was still able to last up to more than 120 h, which was helpful to the wound healing.

The presence of excess ROS in chronically infected wounds severely hinders the normal healing process. Because of its natural enzyme-like properties, MXene can effectively catalyze the decomposition of H_2_O_2_ into H_2_O and O_2_ (Ren et al., 2019; Feng et al., 2021). We used H_2_O_2_ as the demo specie of ROS. After the Cu(II)@MXene incubated with H_2_O_2_ solution for different time, UV-vis was applicated to test the solution absorption in 408 nm (Supplementary Figure S12). In the quantitative analysis (Figure 4K), during the first 120 min, the ROS elimination ratio was quite similar in these three groups [HA hydrogel, MXene hydrogel, and Cu(II)@MXene hydrogel]. It was because that the HA network reacted with H_2_O_2_ firstly to play the main role in the beginning (Stern et al., 2007). Whereafter, as H_2_O_2_ seeped into hydrogel, doped Cu(II)@MXene began to scavenge H_2_O_2_. Compared to pure HA hydrogel, the ROS scavenging ability of Cu(II)@MXene hydrogel was improved by 25%, indicating a good amelioration of oxidative stress microenvironment in infected wounds. The photothermal property of Cu(II)@MXene hydrogel was also tested. Although compared to free Cu(II)@MXene solution, the heating rate of Cu(II)@MXene hydrogel slowed down, it still met the antibacterial requirements for our research (Supplementary Figure S13).

### *In vitro* and *in vivo* characterization of Cu(II)@MXene hydrogels

**FIGURE 5:**
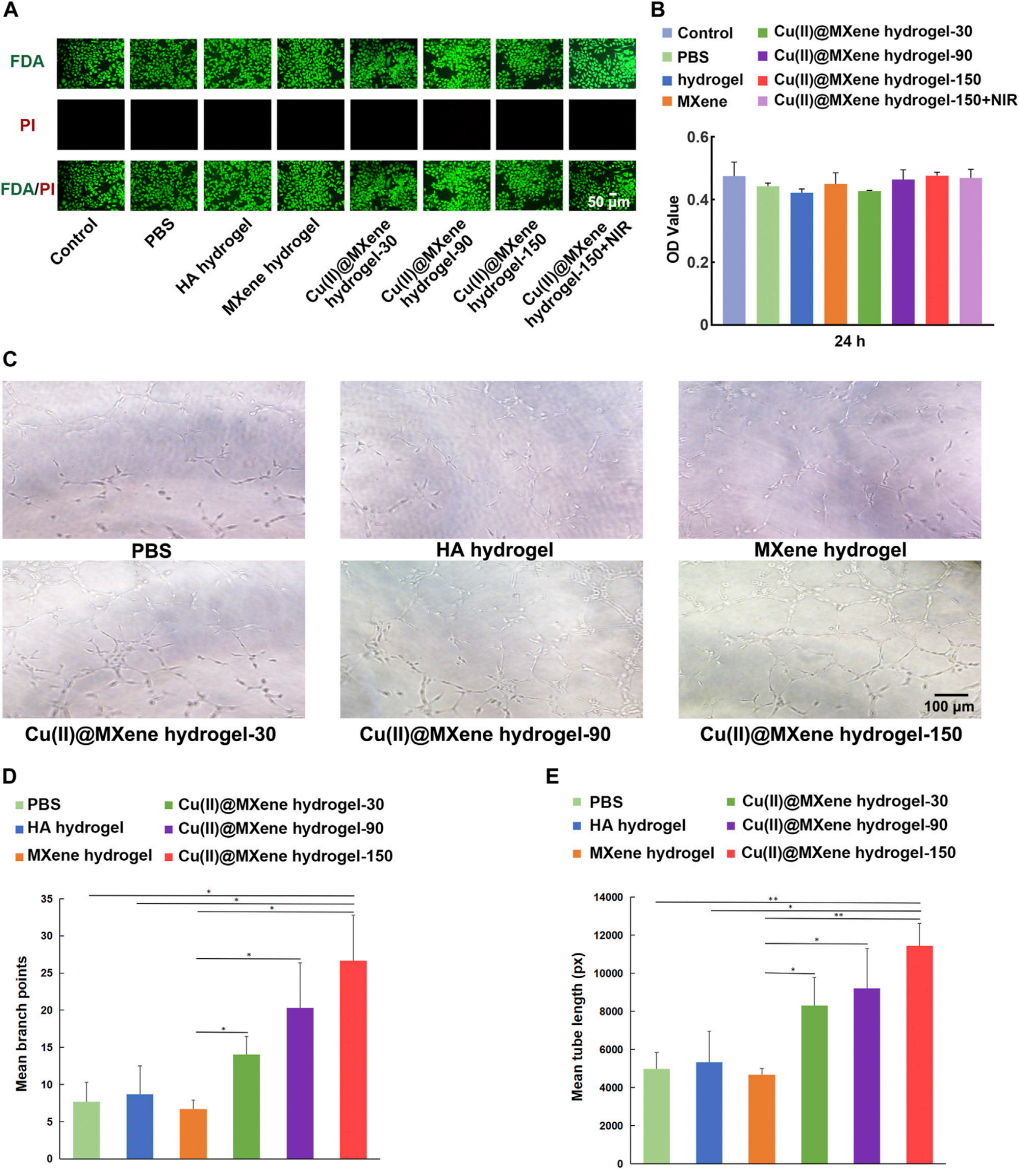
*In vitro* cytocompatibility and angiogenesis property of Cu(II)@MXene hydrogel. **(A)** Live/dead staining images of the L929 cells after being treated with different materials for 24 h. **(B)** CCK-8 results of the L929 cells after treatment with different materials for 24 h. **(C)** Tube formation assay of HUVECs treated by different materials. The quantitative analysis of formed **(D)** branch points and **(E)** tube length, respectively. Statistic results: *p < 0.05. **p < 0.01. ***p < 0.001.

The L929 mouse fibroblast cell lines was cultured with the Cu(II)@MXene hydrogels to evaluate the cytocompatibility. The Live/dead staining images (Figure 5A) showed that all the groups had a satisfactory cell viability. This was also proved by the CCK-8 quantitative analysis (Figure 5B). All of these data demonstrated that our Cu(II)@MXene hydrogel had good biocompatibility. Furthermore, the NIR exposure (1.5 W/cm^2^, 5 min, 45°C) did not show any negative effect on the cell viability (Figure 5B). Angiogenesis is a vital process during the wound healing (Shao et al., 2023). However, endothelial cell dysfunction usually occurred under oxidative stress. To assess the role of Cu(II)@MXene hydrogel in angiogenesis, *in vitro* tube formation assays were obtained by co-culturing HUVECs with hydrogel extract on Matrigel substrates. As shown in Figures 5C–E, the tube formation was not obvious in PBS control group, HA hydrogel group, and MXene hydrogel group. In contrast, these three Cu(II)@MXene hydrogel groups showed a noticeable promoted trend of tube formation through the increase of the Cu(II)@MXene loading concentration. The quantitative analysis (Figures 5D, E) showed that the mean branch points and mean tube length in Cu(II)@MXene hydrogel group (150 μg/mL) was 3.5-fold and 2.3-fold higher than these in the PBS control group, respectively. These results suggested that the Cu^2+^ release from Cu(II)@MXene hydrogel could play a vital facilitation role in angiogenesis in wound healing.

**FIGURE 6:**
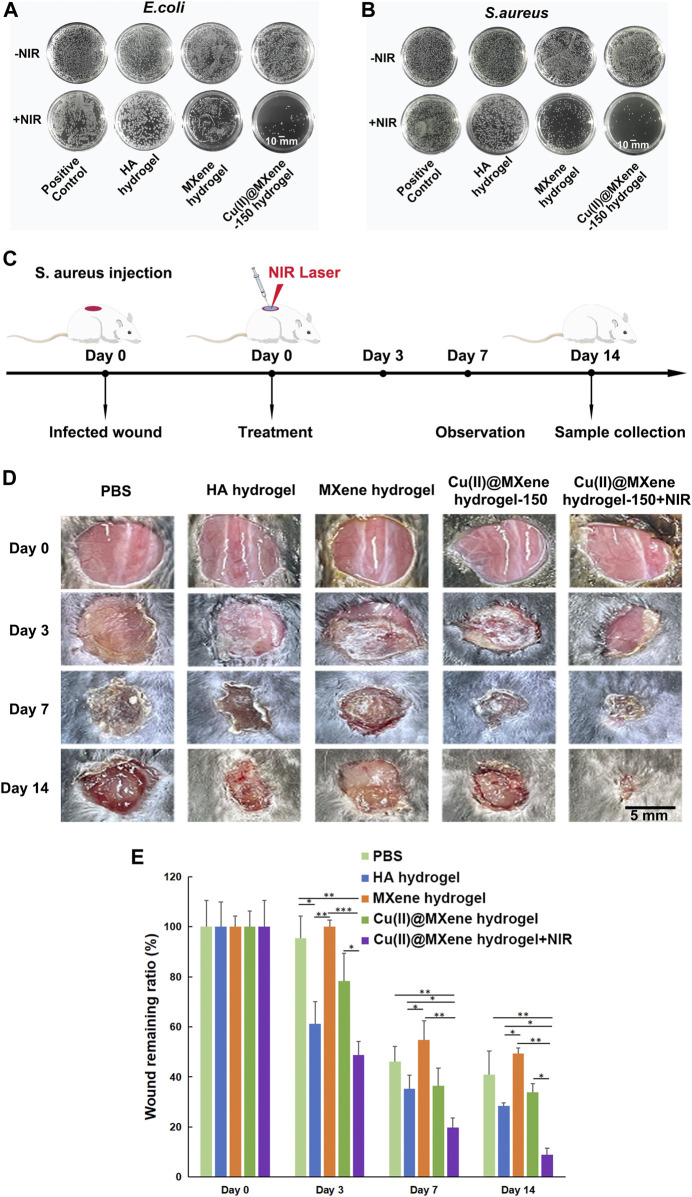
Antibacterial properties of Cu(II)@MXene hydrogel and promotion of infected wound healing *in vivo*. Total view of bacterial colonies formed by *E. coli* **(A)** and *S. aureus* **(B)** after different hydrogel treatments. **(C)** Schematic diagram of the construction of mouse skin infection model. **(D)** Representative images of the wounds at a given time. **(E)** Wound remaining ratios after treatment with different materials. Statistic results: *p < 0.05. **p < 0.01. ***p < 0.001.

The photothermal antibacterial efficiency was evaluated by using *E. coli*, which represented for Gram-negative bacteria, and *S. aureus*, which represented for Gram-positive bacteria. The hydrogels were co-cultured with bacteria with or without NIR irradiation for 5 min, and then after another 12 h of culture. As shown in Figures 6A, B, the MXene hydrogel that only had photothermal effect (45°C) showed a quite low antibacterial effect. As for the Cu(II)@MXene hydrogel + NIR group, the antibacterial efficiency based on the collaboration of photothermal effect and released Cu^2+^ (Supplementary Figure S14) was obvious for *E. coli* and *S. aureus*. The OD_600_ value of the bacterial suspension (Supplementary Figure S15) was also tested to confirm that the antibacterial efficiency of the Cu(II)@MXene hydrogel under NIR irradiation could reach to about 80%. Considering that the concentration of Cu(II)@MXene was fixed at a low level, this result should be satisfactory. After this, an infected wound model of C57BL/6 mice was established for *in vivo* evaluation of wound healing (Figure 6C). Just as we suspected, the Cu(II)@MXene hydrogel NIR groups had the best wound healing efficiency among these four groups after 2 weeks (Figures 6D, E). Compared to Cu(II)@MXene hydrogel group, the wound closure rate increased about 30% in Cu(II)@MXene hydrogel + NIR group, indicating the importance of the bacteria removing during the wound healing.

**FIGURE 7:**
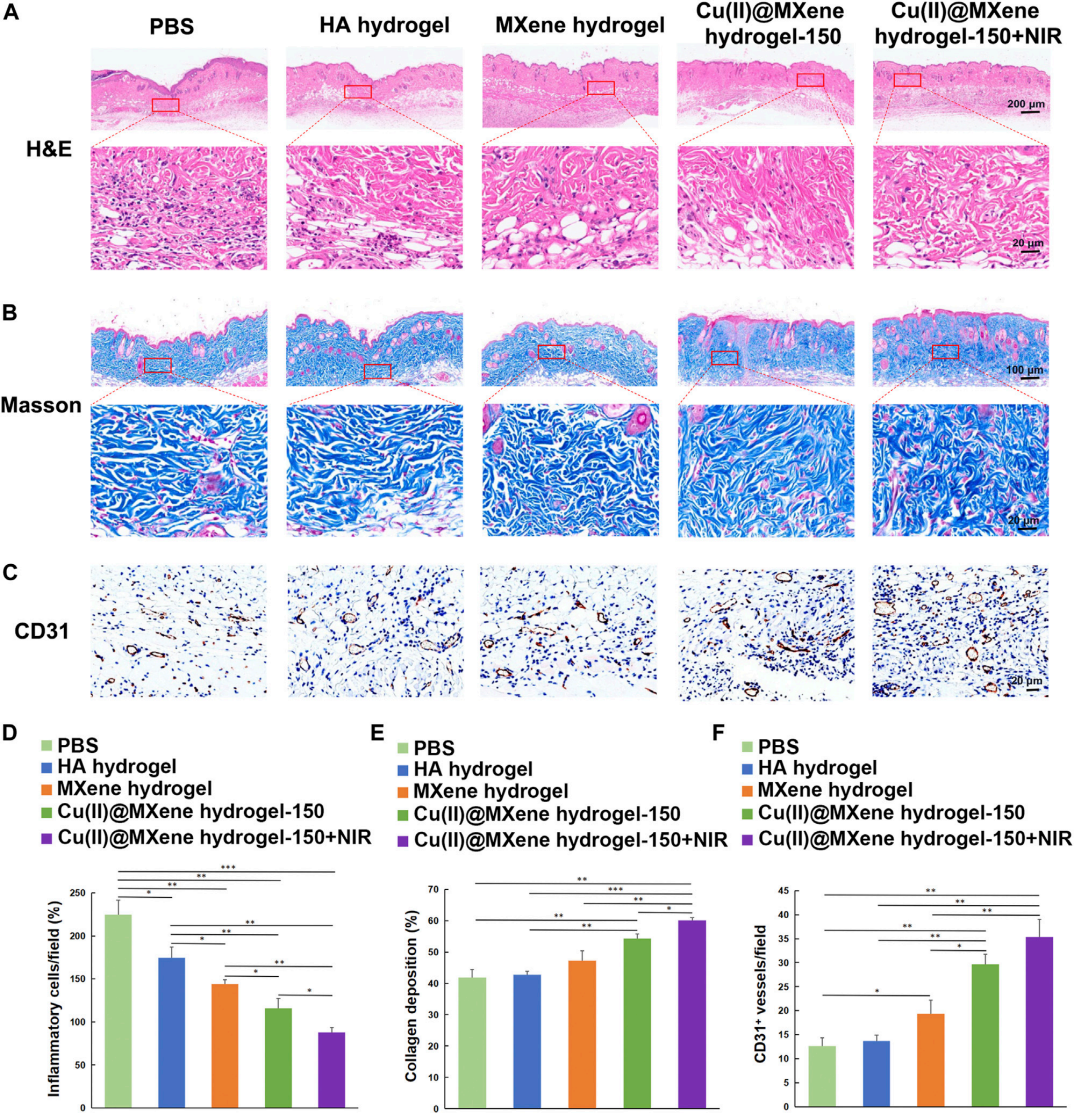
Histologic analysis of the wound tissues. **(A)** H&E and **(B)** Masson’s trichrome staining of wound tissues of the five groups at the day 14. **(C)** IHC images of wound tissues stained for CD31. Quantitative analysis of the **(D)** inflammatory condition based on H&E staining, **(E)** collagen deposition on Masson’s trichrome staining, and **(F)** vessel formation based on CD31 IHC staining. *p < 0.05. **p < 0.01. ***p < 0.001.

Firstly, the collected skin samples were analyzed by H&E staining to observe the status of the neo-epidermis and the inflammatory condition. As shown in Figure 7A, after Cu(II)@MXene hydrogel and NIR treatment, the epithelialization in wound was more complete than the other three groups. Furthermore, fewer inflammatory cells were found in the neo-epidermis of all the MXene hydrogel, Cu(II)@MXene hydrogel, and Cu(II)@MXenehydrogel + NIR group than that in Control group and HA hydrogel group (Figures 7A, D; Supplementary Figure S16). This was because that the ROS removal by MXene was of great help to reduce the inflammation in the wound. From the Masson’s trichrome staining, we further analyzed the collagen deposition in the regeneration skin tissue, with the Cu(II)@MXene hydrogel + NIR group showing the highest degree (Figures 7B, E; Supplementary Figure S17). Section staining of the wound edges further revealed differences in regenerated blood vessels and collagen in the new skin tissue (Supplementary Figures S18, S19). Meanwhile, both the Cu(II)@MXene hydrogel group and Cu(II)@MXene hydrogel + NIR group showed a stronger IHC staining of CD31, a marker for vessel regeneration, than the other groups (Figures 7C, F; Supplementary Figure S20), demonstrating the angiogenesis promopted by the Cu^2+^ release. All of these results proved our Cu(II)@MXene photothermal hydrogel system influenced every step in the infected wound healing positively.

## Conclusion

In short, we successfully prepared a kind of Cu(II)@MXene nanosheet and then constructed the injectable, self-healing, and bio-adhesive Cu(II)@MXene photothermal hydrogel as the dressing for infected wounds. These properties allowed our hydrogels provide long-term protection for wounds. Moreover, after covering the wounds, this Cu(II)@MXene hydrogel effectively remove bacteria by its photothermal conversion under NIR. Meanwhile, this Cu(II)@MXene hydrogel scavenged the excess ROS to reduce inflammation and released the Cu^2+^ to promote angiogenesis. In this way, the Cu(II)@MXene hydrogel could rebuild a favorable microenvironment in infected wounds to accelerated the skin regeneration.

## Data Availability

The original contributions presented in the study are included in the article/Supplementary Material, further inquiries can be directed to the corresponding authors.
